# Spinal Cord Infarction Following Zoledronic Acid Administration in a Patient With Metastatic Prostate Cancer

**DOI:** 10.7759/cureus.96011

**Published:** 2025-11-03

**Authors:** Brittany E Reid, Mario Perez, Zakaria Abd Elmageed, Stephen DiGiuseppe, Katharine Balbuena

**Affiliations:** 1 Rehabilitation Medicine, Independent Researcher, Chicago, USA; 2 Research, Independent Researcher, Mobile, USA; 3 Pharmacology, Edward Via College of Osteopathic Medicine, Monroe, USA; 4 Microbiology, Edward Via College of Osteopathic Medicine, Monroe, USA; 5 Physical Medicine and Rehabilitation, Willis Knighton Rehabilitation, Shreveport, USA

**Keywords:** bisphosphonates, metastatic prostate cancer, spinal cord infarction, spinal cord injury, zoledronic acid

## Abstract

Spinal cord infarction is a rare but serious complication that can occur in the setting of metastatic disease, prior radiation therapy, and vascular compromise. We present a case of a 69-year-old male who was admitted to inpatient rehabilitation following an acute spinal cord infarction. Past medical history included metastatic prostate cancer, osteoporosis, deep vein thrombosis on apixaban, and a complex oncologic and spinal history. He experienced a sudden onset of back pain that progressed to bilateral leg paralysis and bowel and bladder dysfunction. His symptoms presented within 24 hours of receiving intravenous zoledronic acid for metastatic bone disease. Imaging revealed T2 signal changes suggestive of spinal cord infarction and new syringohydromyelia. Neurology suspected ischemia of the artery of Adamkiewicz in the context of metastatic disease, prior radiation, and bisphosphonate exposure. During inpatient rehabilitation, the patient demonstrated gradual motor recovery, improved sitting balance, and increased functional independence with therapy, despite experiencing persistent bowel and bladder incontinence. He developed a urinary tract infection and later hematuria, which required hospital readmission. He was then discharged to a skilled nursing facility for continued rehabilitation. This case highlights an uncommon but devastating neurologic complication in the context of complex oncologic care and prompts the consideration of potential vascular risks of bisphosphonates in patients with prior spinal metastases, radiation, and surgery. It also emphasizes the diagnostic complexity and the potential for rehabilitation in spinal cord infarction with multifactorial oncologic and vascular compromise.

## Introduction

Spinal cord infarction (SCI) is a rare type of stroke that can develop from various etiologies and accounts for 0.3-1% of strokes [1,2]. The rarity of SCI is potentially due to the abundance of collateral circulation between the anterior and posterior spinal arteries [1,3,4]. In the adult population, the most common causes are atherosclerosis, aortic surgeries, and thoracic aortic aneurysm [2]. According to one study, cardiovascular risk factors such as hypertension, diabetes, and smoking account for 44.2% of SCI cases [1], consistent with other reports identifying them as the leading etiologies [3,5]. Hypertension alone accounts for 17.1% of cases, trauma to the spine accounts for 14.3%, and a third of cases had no known etiology [1]. Among iatrogenic causes, patients undergoing thoracoabdominal aortic surgery have an SCI risk of up to 33% [3], while other iatrogenic causes (epidural anesthesia or spinal anesthesia) do occur, but their overall prevalence was not reported [3,6]. Infrequent causes of SCI include hypercoagulable states and occlusions of the artery of Adamkiewicz [4]. Bisphosphonate use has not previously been implicated as a cause of SCI.

Occlusion of the artery of Adamkiewicz is another recognized cause of SCI [7,8]. The artery of Adamkiewicz arises around the T8 to L2 level and, as a result, provides the bulk of the blood supply to the lower thoracic and lumbar portions of the anterior spinal cord [3,7]. Anatomic variations may allow this artery to originate as high as T3. Occlusion of the artery of Adamkiewicz typically results in anterior cord syndrome, characterized by bilateral motor paralysis, bowel and bladder dysfunction, and loss of pain and temperature sensation below the level of infarction [7,8].

While SCI can present with a wide range of clinical features, patients initially report back pain or radicular pain localized to the level of injury [1,6]. Unlike strokes, the presentation of SCI is prolonged rather than acute [1,4]. Symptoms can occur within 24 hours, but most are present within six hours. Patients who had symptoms within 12 hours were more likely to be suffering from SCI at the thoracic level, while patients with symptoms within 72 hours had an SCI at the cervical level. Neurological symptoms such as hemiplegia (paralysis of left or right extremities), paraplegia (paralysis of either upper or lower extremities), tetraplegia (paralysis of all extremities), loss of pain sense, loss of temperature sense, and bladder atony can occur depending on the level of the infarction [1,2].

Diagnosis of this condition requires a thorough history, physical exam, and prompt medical imaging. The gold standard for evaluating suspected SCI is magnetic resonance imaging (MRI), particularly T1-, T2-, and diffusion-weighted imaging (DWI) [1,3]. In the hyperacute stage (<12 hours from onset), MRI is not very sensitive [1,9]. Within the acute phase (<24 hours), DWI is becoming more sensitive to the changes from SCI [3,9]. There are a few "classic" signs on MRI, but they are not specific to this condition [1,3]. On T2-weighted imaging (T2WI), spinal cord swelling and "pencil-like" hyperintensities are expected. Another characteristic finding is the presence of "owl's eyes" on axial T2WI. Post-contrast T1-weighted imaging (T1WI) may demonstrate enhancement, and DWI often reveals a hyperintense signal [3]. If the initial MRI is normal, then a repeat MRI should be ordered [6]. Most lesions (up to 92.5%) occur at the T8-L2 and C4-T4 levels [9].

Treatment for SCI should be tailored to the suspected cause. If patients suffer from cardiac embolism or coagulopathies, they should start direct-acting oral anticoagulants (DOAC). It is recommended to add steroids to the treatment plan of patients who suffer from vasculitis [3]. Regardless of etiology, aggressive physical therapy has been shown to help in recovery [3,4].

Herein, we present a rare case of SCI in a patient with prevalent risk factors for SCI, such as malignancy-related hypercoagulability, hyperlipidemia, and several iatrogenic causes, including recent bisphosphonate use. Although SCI accounts for only 0.3-1% of strokes, it carries significant morbidity and is often underrecognized due to its variable presentation and overlap with other spinal pathologies. The possible infarction of the artery of Adamkiewicz, with associated syringohydromyelia, further underscores the diagnostic complexity. Earlier recognition may have been possible with higher suspicion for vascular causes in the setting of acute urinary retention, rapid neurologic decline, and non-compressive MRI findings. This case also emphasizes the importance of carefully considering bisphosphonate use in complex patients with many overlapping risk factors.

## Case presentation

A 69-year-old male presented to inpatient rehabilitation after visiting the emergency department (ED) for sudden leg weakness. On admission to the ED, the patient reported experiencing left leg weakness that began that morning. His past medical history included osteoporosis, venous insufficiency, metastatic castration-sensitive adenocarcinoma of the prostate with antineoplastic chemotherapy status post prostatectomy, osteolytic lesion due to metastasis, anemia, osteoarthritis, pulmonary embolus, and deep vein thrombosis on apixaban (Eliquis®). He stated that he went to the bathroom and then experienced sharp lower back pain. Then he began to feel off balance while walking back to his bed. Once in bed, he experienced increasing weakness until he was unable to move his left leg, which initiated his visit to the ED.

In the ED, he denied any other symptoms, recent falls, or recent trauma. The patient was diagnosed with localized prostate cancer in 2016, nine years prior to this admission. He underwent radical prostatectomy and radiation for recurrence and later presented in 2023 with symptomatic metastatic disease in the thoracic spine. His treatment course included spinal surgery, radiation therapy, and systemic therapy with androgen deprivation, abiraterone, and docetaxel chemotherapy. Shortly after completing chemotherapy, he received a scheduled dose of zoledronic acid (5 mg infusion) for metastatic bone disease and osteoporosis. He reported receiving intravenous zoledronic acid the day prior to his onset of symptoms. Past surgical history included thoracic spinal fusion and prostatectomy. Family history included heart disease and stroke. His social history was significant for being a former smoker, at the rate of 13 packs per year for 20 years; his quit date was over 15 years prior to the time of admission. He reported consuming one to two alcoholic drinks per day, either beer or hard liquor, and denied illicit drug use. The patient had no known drug allergies. The patient's home medications included abiraterone 1,000 mg once per day, apixaban 2.5 mg twice per day, aspirin 325 mg once per day, atorvastatin 40 mg at bedtime, docusate sodium 100 mg twice per day, and prednisone 5 mg two times per day.

Review of systems was negative for any other symptoms. Vitals in the ED were notable for a temperature of 96℉ and a blood pressure of 137/93 mmHg. Neurosurgery and neurology evaluations showed Hoffman's sign on the left. No clonus was found, but the lower extremities were 0/5 for strength with hyporeflexia. There was no temperature sensation below T4; however, sensation to light touch and toe proprioception was normal. He was positive for urinary retention. While in the ED, he started to experience right lower extremity weakness as well.

Laboratory results on ED admission were normal other than the findings in Table 1; his hemoglobin was 11.6 g/dL (reference range: 13.1-16.8 g/dL), International Normalized Ratio (INR) 1.1 (reference range: 0.8-1.2), B-Natriuretic Peptide 381 pg/mL (reference range: 15-100 pg/mL), potassium 3.1 mmol/L (reference range: 3.4-5.1 mmol/L), and calcium 7.7 mg/dL (reference range: 8.6-10.3 mg/dL). Cerebrospinal fluid (CSF) results from the hospital admission prior to rehabilitation (Table 1) showed a hazy appearance, 3355 red blood cells (RBC) # cells (reference range: 0-10/cu.mm), glucose 85 mg/dL (reference range: 40-70 mg/dL), and protein 141 mg/dL (reference range: 12-60 mg/dL). Imaging on the day of admission to the ED revealed widespread degenerative and metastatic changes without acute compressive pathology. Computed tomography (CT) of the head, neck, chest, and lumbar/thoracic spine showed age-related changes, no acute intracranial or vascular abnormalities, and severe multilevel lumbar and thoracic spondylosis with metastatic lesions. MRI of the lumbar and thoracic spine confirmed extensive degenerative disc disease, multilevel stenosis, and sclerotic vertebral lesions.

**Table 1:** Laboratory results from ED admission

Test	Value	Reference Range
Hemoglobin	11.6 g/dL	13.1–16.8 g/dL
B-Natriuretic Peptide	381 pg/mL	15–100 pg/mL
Potassium	3.1 mmol/L	3.4–5.1 mmol/L
Calcium	7.7 mg/dL	8.6–10.3 mg/dL
International Normalized Ratio (INR)	1.1	0.8–1.2
CSF Red Blood Cells (RBC) # Cells Count	3355/cu.mm	0–10/cu.mm
CSF Protein	141 mg/dL	12–60 mg/dL
CSF Glucose	85 mg/dL	40–70 mg/dL

**Figure 1:**
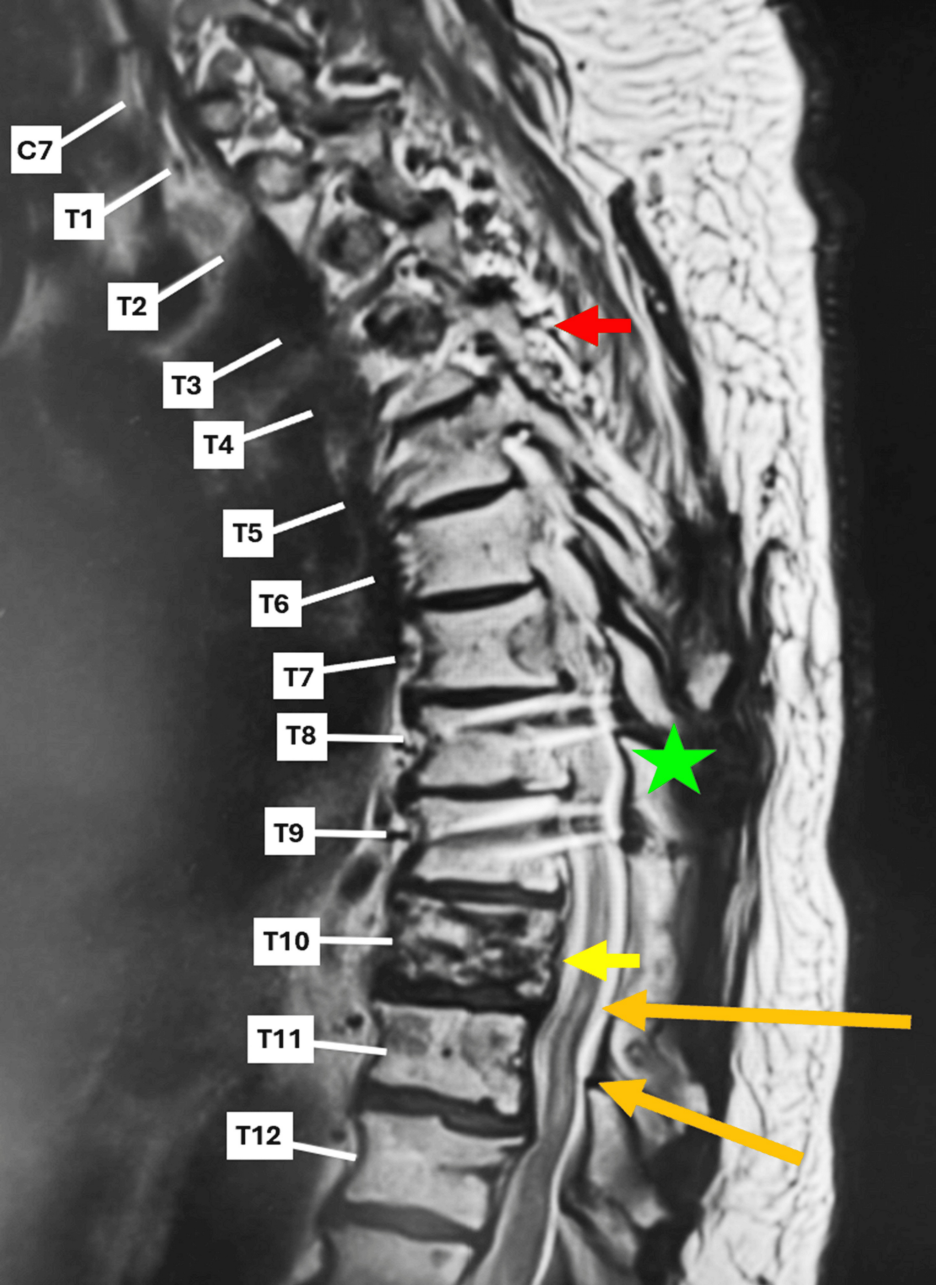
Thoracic spine MRI taken on ED admission shows postoperative changes T-spine MRI showing postoperative changes from the laminectomy at T10 (orange arrows), changes from transpedicular fusion at T8-12 (green star), T2 hyperintensity in the spinal cord from T3 (red arrow) to T7, and multiple metastatic deposits present in the thoracic spine, especially at T10 (yellow arrow).

Initial T-spine MRI showed postoperative laminectomy changes at T10, transpedicular fusion changes at T8-12, metastatic deposits at T10, and T2 hyperintensity in the spinal cord from T3 to T7, raising concern for SCI or inflammatory myelopathy (Figure 1). It was noted that there was a possible artery of Adamkiewicz infarction and new syringohydromyelia from T5 to T7.

**Figure 2:**
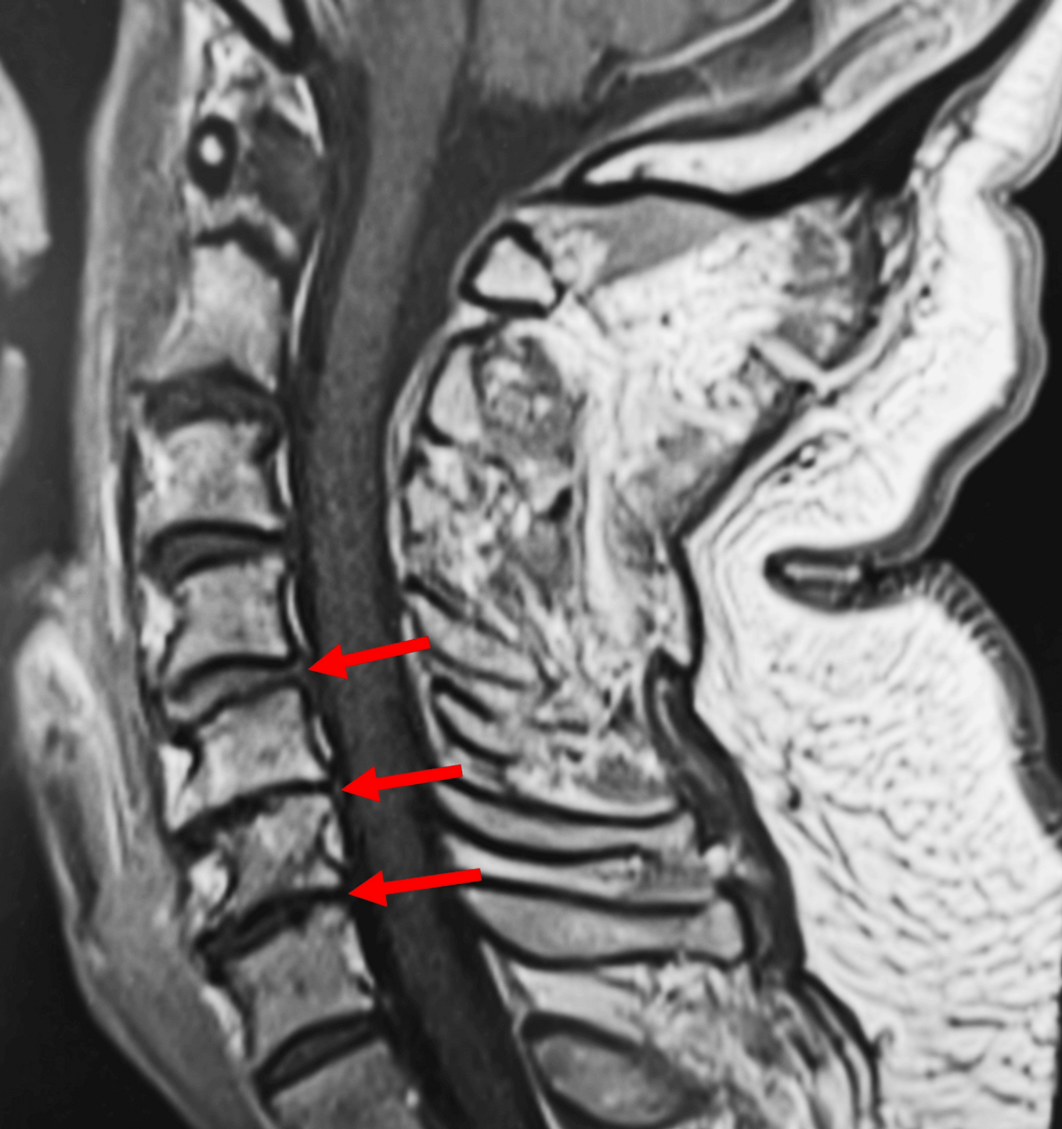
Cervical spine MRI on day two of hospital admission showed degenerative disc disease A cervical MRI on day two of hospital admission showed degenerative disc disease at C3-C6 (red arrows), but normal cord signal intensity.

A cervical spine MRI on day two of hospital admission showed multilevel degenerative changes in C3-C6 without a cord signal abnormality (Figure 2). A computed tomography angiography (CTA) of the abdomen and pelvis ruled out vascular occlusion of the celiac, superior mesenteric, and inferior mesenteric arteries. An MRI of the brain was also negative for acute ischemia. A fluoroscopy-guided lumbar puncture yielded limited CSF and was difficult to perform due to spinal stenosis. X-ray of the kidneys, ureters, and bladder (KUB) showed moderate stool burden but no acute abdominal pathology. Overall, imaging supported the diagnosis of SCI in the setting of known metastatic spine disease, prior radiation, and bisphosphonate therapy. Neurology suspected an occlusion of the artery of Adamkiewicz and diagnosed the patient with an SCI. They determined he would be a candidate for inpatient rehabilitation since he was previously fully independent and living alone.

On his rehabilitation admission day, his review of systems was positive for diarrhea, fecal incontinence, urinary retention, back pain and bilateral leg paralysis, muscle cramps, inability to walk, and skin color changes in both legs from venous insufficiency. Vital signs on rehabilitation admission were relatively normal, with a temperature of 98.0℉, a pulse of 69 beats per minute, respirations of 14 per minute, blood pressure of 129/74 mmHg, and oxygen saturation of 99% on room air. Physical examination revealed a soft, non-tender abdomen with minor distension and hypoactive bowel sounds. A urinary catheter was inserted for urinary incontinence. He had general back tenderness on examination, and he reported mild chronic back pain due to his previous spinal issues. An old deep-tissue injury to the coccyx, which was healing, was also present on examination. Sensory was intact, and normal muscle tone was noted. The patient endorsed paresthesia in the legs and feet. Motor testing was 0/5 bilaterally for the legs; however, it was difficult to isolate and test the thoracic level due to patient limitations. A skin exam revealed dry skin, especially on the feet, and the legs were firm with dark venous changes, although there were no lesions present.

Laboratory results on rehabilitation hospital admission (Table 2) were unremarkable other than a hemoglobin of 12 g/dL (reference range: 13.1-16.8 g/dL), calcium of 7.6 mg/dL (reference range: 8.6-10.3 mg/dL), and 25-hydroxy vitamin D <12.8 ng/mL (reference range: 30.0-100.0 ng/mL).

**Table 2:** Laboratory results from the rehabilitation hospital admission

Test	Value	Reference Range
Hemoglobin	12 g/dL	13.1–16.8 g/dL
Calcium	7.6 mg/dL	8.6–10.3 mg/dL
25-Hydroxy Vitamin D	<12.8 ng/mL	30–100 ng/mL

Physical therapy started on the third day of rehabilitation, and the patient was seen in the gym with low but stable blood pressure and no syncope and progressed to sitting balance exercises. A Foley catheter was removed despite absent bladder sensation, as he demonstrated spontaneous voiding without evidence of retention. A bowel regimen was initiated concurrently. By the fourth day, he exhibited improved sitting balance, allowing progression to slide board transfers with physical therapy, though he continued to require a Hoyer lift (Joerns Healthcare, Charlotte, North Carolina) for nursing transfers.

In the subsequent days, gradual motor recovery was observed, including gluteal and quadriceps activation during assisted standing activities. On the sixth day, he developed new labile hypertension without prior history, prompting an evaluation for autonomic dysreflexia. A bladder scan was obtained, and urinalysis with culture revealed an *Escherichia coli* (*E. coli*) urinary tract infection, for which cefpodoxime (100 mg PO q12hr for four to seven days) was initiated based on antibiotic sensitivities for the *E. coli* strain. Therapy sessions continued with noted gains in mobility and coordination; however, persistent bowel and bladder incontinence presented significant barriers to progress. A timed straight catheterization schedule was implemented, which reduced urinary accidents during therapy. Despite these efforts, his constipation continued, and his bowel program was intensified with scheduled regimens and the addition of lactulose.

Care planning shifted toward replicating home routines, including morning bowel care, and co-treatment with occupational therapy. Discussions about long-term bladder management were held, including the possibility of Foley reinsertion, especially in the context of his prior prostatectomy and probable neurogenic bladder. Oncology follow-up occurred during admission, with hormonal therapy placed on hold. By day 18, he demonstrated increasing motor control, particularly in hip mobility and postural alignment, and the family began considering discharge to live with his son. He achieved supervised-level assistance for bed mobility and minimal assistance for quadruped transfers. While the patient voiced frustration with ongoing incontinence, his therapy led to measurable improvements in strength, coordination, and functional independence. Three weeks after rehabilitation admission, the patient developed hematuria when he self-catheterized and reported some bladder discomfort. His Foley also revealed many clots, so he was transported from the rehabilitation hospital to the main hospital. After 10 days in the main hospital, he was discharged to a skilled nursing facility for further rehabilitation.

## Discussion

The patient described in this report had several risk factors, such as cardiovascular risk factors (history of tobacco use and venous insufficiency), hypercoagulable state secondary to metastatic adenocarcinoma of the prostate, and several iatrogenic risk factors (thoracic surgery, chemotherapy, and zoledronic acid). What makes this case more interesting is that most of these risk factors are temporally distant from the onset of the SCI. The patient's risk factors and history are key to SCI diagnosis because MRI findings can resemble those of other neurological conditions, and the findings ultimately depend on where the damage occurs, not which risk factor led to the neurological insult [9,10].

As cardiovascular risk factors are the leading cause of SCI, they are worth discussing first. There are no established blood pressure targets specifically recommended for the prevention of SCI. As such, risk assessment can be extrapolated from the patient's atherosclerotic cardiovascular disease (ASCVD) risk score. Based on his clinical variables, the ASCVD calculator placed this patient in the intermediate-risk category, corresponding to a 7.5-19.9% likelihood of myocardial infarction or stroke within the next 10 years [11]. Given this, he remains at risk for stroke and thus for an SCI; however, his use of a hydroxymethylglutaryl-CoA reductase inhibitor (atorvastatin) has been shown to provide protective benefits in reducing stroke risk [11].

The patient's history of cancer could be a potential risk factor as well. Most cancers cause hypercoagulable states or raise the risk of thrombotic events due to chemotherapy [12]. Cancer patients are most at risk for venous thromboembolic events, specifically with a four- to nine-fold risk compared to the general population [12]. This is exemplified in our patient, who had a history of deep vein thrombosis (DVT) that resulted in a pulmonary embolism. Cancer patients are additionally at risk for several other complications, including arterial thrombosis, nonbacterial thrombotic endocarditis, superficial thrombophlebitis, catheter-associated thrombosis, and hepatic veno-occlusive disease [13]. Despite the known embolic risks, only one case to our knowledge has reported cancer-associated hypercoagulability leading to SCI, typically after an initial hospital presentation with pulmonary embolism [13]. The patient in this case was not on a DOAC, but he was started on enoxaparin, a low-molecular-weight heparin (LWMH), and was on day four of taking enoxaparin when he suffered from SCI. DOAC and LWMH have been shown to be effective in preventing these embolic events [5]. In our case, the patient was on apixaban 2.5 mg twice per day, which served as his DOAC.

The occlusion of the artery of Adamkiewicz commonly causes anterior cord syndrome [7,8], which is a common cause of SCI [8]. The risk factors for the artery of Adamkiewicz occlusion include hypertension, dyslipidemia, diabetes mellitus, and smoking [8]. These risk factors are like the risk factors for SCI, although SCI has a higher emphasis on hypertension and smoking [1,3]. Our patient did not have a prior history of hypertension or diabetes mellitus, but he did have a prior history of smoking. If the patient had anterior cord syndrome at the T3-T7 level, where the T2 hyperintensity in the spinal cord was present on imaging, he would not be expected to perform sitting exercises requiring abdominal muscle activation. Taken together, the clinical picture suggests a higher thoracic spinal level SCI than would be expected from the typical presentation of an occlusion of the artery of Adamkiewicz. This interpretation is further supported by MRI findings of syringohydromyelia extending from T5 to T7, which lies above the typical origin of the artery of Adamkiewicz. The most plausible explanation is that the infarction arose from the convergence of multiple risk factors, rather than a single determinant, predisposing the patient to this ischemic event despite most risk factors being appropriately managed.

In terms of iatrogenic causes, the medications treating the patient's prostate cancer, including degarelix, leuprolide, and abiraterone, are known risk factors for major adverse cardiovascular events (MACE) such as myocardial infarction, acute coronary syndrome, strokes, and cardiovascular death [14-16]. These events typically occur within the first four months of therapy for degarelix and leuprorelin [17]. Our patient's last dose of degarelix was given four years prior to his SCI. He was last treated with leuprorelin five months prior to his SCI. Both fall outside the initial four-month window, but degarelix and leuprorelin may still have contributed to the SCI event. He was prescribed abiraterone and prednisone after his last leuprorelin dose. While abiraterone has been associated with increased MACE, pairing abiraterone with a steroid may reduce the odds of these events [17]. There is some debate regarding whether statins and other preventative cardiovascular measures can reduce the risk of MACE [14]. We suggest that using steroids with abiraterone and having the patient on apixaban helped reduce the likelihood of a MACE.

Our patient was diagnosed with adenocarcinoma of the prostate with metastasis to T10. He had a T8-T10 spinal fusion with excision of the T10 extradural tumor. He subsequently had radiation therapy from T6 to T12 along with chemotherapy (degarelix) and was in treatment with an abiraterone and steroid combination. This surgical history may have predisposed him to an SCI, since the T2 hyperintensity on MRI was affecting the T3-T10 level. Prior history of spinal surgery was a risk factor in a few studies [1,2,5]. This surgery occurred almost two years prior, so it may not have been an immediate cause, but it could be a strong risk factor based on the area that was operated on and where our patient had their hyperintensity MRI findings.

The patient was treated with a 5 mg infusion of zoledronic acid for osteoporosis. Although there is ongoing debate about the effects of bisphosphonates on atherosclerotic plaques, current evidence suggests that this drug class does not destabilize plaques. The atherosclerotic effects of bisphosphonates are mostly seen in small observational studies and animal studies [18]. The timing of the infusion raises the possibility that it served as a precipitating factor for the patient's SCI, as symptoms developed within 24 hours of administration. One alternative consideration is a venous air embolism; however, this seems less likely in the absence of a patent foramen ovale or an occult fistulous tract. In the event of an air embolism, the gas would typically remain confined to the venous circulation and lodge in the pulmonary vasculature, unless it were able to cross into the arterial system via a right-to-left shunt, patent foramen ovale, or intrapulmonary arteriovenous malformations [19,20]. Since our patient had none of these conditions, the likelihood of an arterial air embolism is very low.

## Conclusions

This case highlights the complexity of SCI in patients with metastatic prostate cancer, prior radiation therapy, spinal surgery, and bisphosphonate use. The temporal relationship between zoledronic acid infusion and symptom onset raises consideration of a potential contributing role, though causality cannot be established without structured analysis. Clinicians should maintain vigilance for new neurological symptoms following infusion in high-risk patients. Early recognition, initiation of rehabilitation, and close follow-up remain critical for optimizing functional recovery and outcomes.

## References

[1] Dokponou YC, Ontsi Obame FL, Takoutsing B (2024). Spinal cord infarction: a systematic review and meta-analysis of patient's characteristics, diagnosis accuracy, management, and outcome. Surg Neurol Int.

[2] Romi F, Naess H (2016). Spinal cord infarction in clinical neurology: a review of characteristics and long-term prognosis in comparison to cerebral infarction. Eur Neurol.

[3] Hanna Al-Shaikh R, Czervionke L, Eidelman B, Dredla BK (2025). Spinal cord infarction. StatPearls [Internet].

[4] Zalewski NL, Rabinstein AA, Krecke KN (2019). Characteristics of spontaneous spinal cord infarction and proposed diagnostic criteria. JAMA Neurol.

[5] Gharios M, Stenimahitis V, El-Hajj VG (2024). Spontaneous spinal cord infarction: a systematic review. BMJ Neurol Open.

[6] Pigna F, Lana S, Bellini C, Bonfanti L, Creta M, Cervellin G (2021). Spinal cord infarction. A case report and narrative review. Acta Biomed.

[7] Lindeire S, Hauser JM (2025). Anatomy, back, artery of Adamkiewicz. StatPearls [Internet].

[8] Sandoval JI, De Jesus O (2025). Anterior spinal artery syndrome. StatPearls [Internet].

[9] Ke G, Liao H, Chen W (2024). Clinical manifestations and magnetic resonance imaging features of spinal cord infarction. J Neuroradiol.

[10] Yadav N, Pendharkar H, Kulkarni GB (2018). Spinal cord infarction: clinical and radiological features. J Stroke Cerebrovasc Dis.

[11] Goff DC Jr, Lloyd-Jones DM, Bennett G (2014). 2013 ACC/AHA guideline on the assessment of cardiovascular risk: a report of the American College of Cardiology/American Heart Association Task Force on Practice Guidelines. Circulation.

[12] Ghorbanzadeh A, Porres-Aguilar M, McBane R, Gerotziafas G, Tafur A (2025). Extended anticoagulation in patients with cancer-associated venous thromboembolism. Pol Arch Intern Med.

[13] Thar YY, Tun AM, Huang T, Bordia S, Guevara E (2015). Spinal cord infarct as a presentation of cholangiocarcinoma with metastases. Ann Transl Med.

[14] Lopes RD, Higano CS, Slovin SF (2021). Cardiovascular safety of degarelix versus leuprolide in patients with prostate cancer: the primary results of the PRONOUNCE randomized trial. Circulation.

[15] Serrano Domingo JJ, Alonso Gordoa T, Lorca Álvaro J (2021). The effect of medical and urologic disorders on the survival of patients with metastatic castration resistant prostate cancer treated with abiraterone or enzalutamide. Ther Adv Urol.

[16] Lee YH, Hui JM, Leung CH (2024). Major adverse cardiovascular events of enzalutamide versus abiraterone in prostate cancer: a retrospective cohort study. Prostate Cancer Prostatic Dis.

[17] Melloni C, Slovin SF, Blemings A (2020). Cardiovascular safety of degarelix versus leuprolide for advanced prostate cancer: the PRONOUNCE trial study design. JACC CardioOncol.

[18] Kim DH, Rogers JR, Fulchino LA, Kim CA, Solomon DH, Kim SC (2015). Bisphosphonates and risk of cardiovascular events: a meta-analysis. PLoS One.

[19] Shin KM, Lim JK, Kim CH (2014). Delayed presentation of cerebellar and spinal cord infarction as a complication of computed tomography-guided transthoracic lung biopsy: a case report. J Med Case Rep.

[20] He R, Huang Q, Yan X, Liu Y, Yang J, Chen X (2019). A case of paradoxical embolism causing anterior spinal cord syndrome and acute myocardial infarction following the intradiscal oxygen-ozone therapy. Front Neurol.

